# Olfaction-Inspired Sensing Using a Sensor System with Molecular Recognition and Optimal Classification Ability for Comprehensive Detection of Gases

**DOI:** 10.3390/s140305221

**Published:** 2014-03-12

**Authors:** Masahiro Imahashi, Masashi Watanabe, Sunil Kumar Jha, Kenshi Hayashi

**Affiliations:** Department of Electronics, Graduate School of Information Science and Electrical Engineering, Kyushu University, 744 Motooka, Fukuoka 819-0395, Japan; imahashi@o.ed.kyushu-u.ac.jp (M.I.); watanabe@o.ed.kyushu-u.ac.jp (M.W.); sdrsunil76@gmail.com (S.K.J.)

**Keywords:** bio-inspired model, molecular recognition, molecular imprinting, odor material classification, odor map, odor clustering

## Abstract

In this study, we examined the comprehensive detection of numerous volatile molecules based on the olfactory information constructed by using olfaction-inspired sensor technology. The sensor system can simultaneously detect multiple odors by the separation and condensation ability of molecularly imprinted filtering adsorbents (MIFAs), where a MIP filter with a molecular sieve was deposited on a polydimethylsiloxane (PDMS) substrate. The adsorption properties of MIFAs were evaluated using the solid-phase microextraction (SPME) and gas chromatography-mass spectrometry (GC-MS). The results demonstrated that the system embedded with MIFAs possesses high sensitivity and specific selectivity. The digitization and comprehensive classification of odors were accomplished by using artificial odor maps constructed through this system.

## 1. Introduction

Recently, the demands for sensing and digitization of odor information are increasing rapidly. Over the past few decades, odor sensors that use cross-selective sensor arrays, e.g., electronic nose (e-nose) technology, have been actively researched [1,2]. These high-performance sensors can measure the difference between VOCs and classify chemicals by analyzing datasets obtained using conventional instruments and sensor arrays, e.g., chemoresistor or quartz crystal microbalance sensors [3]. Moreover, by adding sensing mechanism inspired by recognition processes of biological olfaction to these sensor array devices, the higher levels of qualitative detection and discrimination of many odorants was realized [3].

Biological olfactory systems extract odor information from odorants using olfactory receptors (ORs) [4,5] and nasal mucosa [6,7]. In particular, several specific ORs corresponding to one odorant are activated [8,9]. Malnic and coworkers have revealed that biological olfaction discriminates odors using different combinations of active signals from ORs, and then individual ORs recognize certain specific molecular properties of odorants, e.g., several consecutive carbon chain lengths and functional groups [10,11].

An odor map is formed in a specifically located glomeruli sheet on the olfactory bulb (OB), where the molecular receiving specificity of ORs is expressed [12–15]. Odor maps become the first internal expressions of odor in the brain [5]. Therefore, it is said that the difference between odor maps has a direct correlation with the difference of odor quality [5,16]. Here, glomeruli with similar molecular specificity are located close to each other. Therefore, odorants with similar molecular features are classified into the same cluster in the odor map [5,11,16–19], which is called “odor clustering”. The biological olfaction can discriminate and optimally classify numerous chemicals by executing such compression processing of odor information [5,16].

**Figure 1.**
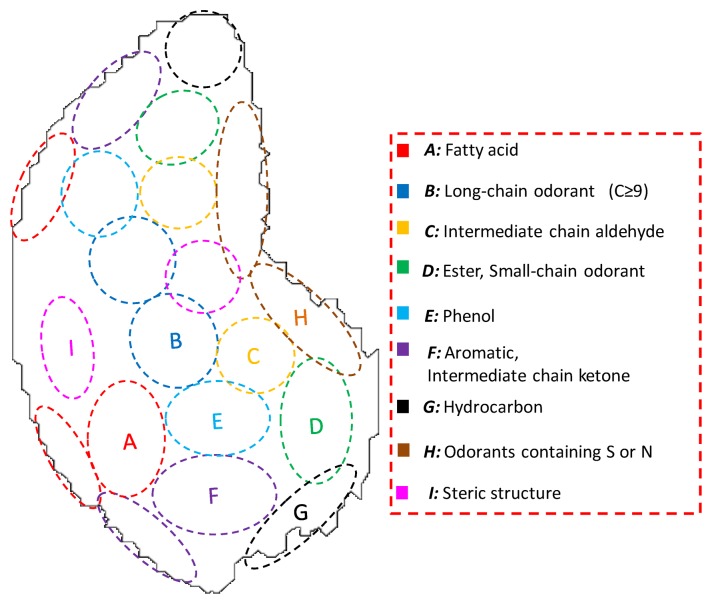
The illustration of odor map and the geometric feature in the biological olfaction based on mapping patterns of rats in the Leon laboratory. Odorants with similar molecular structures are classified into the same cluster.

To apply odor clustering to sensor technology, previous research examined the extraction of the clustering and geometric features of odor maps by analyzing odor map images developed in other laboratories [20]. The defined odor clustering maps and the common characteristics of odorants belonging to each cluster were described in Figure 1. For instance, fatty acids have carboxylic groups, hence, they belong to the cluster A. The biological olfactory enables comprehensive detection and appropriate classification of odors by the discrimination of structural features of odorants described in Figure 1 and optimally classify odorants into clusters based on the ORs signals. Therefore, olfaction-inspired sensing is possible by sensor devices with critical molecular recognition ability that can construct odor-clustering maps of biological olfaction.

We manufactured an odor-separating system which separately measures odors by the combination of different adsorbent materials adhered to microceramic heaters and metal oxide semiconductor gas (MOX) sensors [21–23]. Our previous research has succeeded in estimating the molecular size and polarity of odorants using the system that applied carbon molecular sieves and gas chromatography column adsorbent materials [22]. This system realized molecular features extraction of odorants by the addition of molecular recognition parts into cross-selective sensor arrays. In addition, the odor-clustering maps were constructed, while the precision was not satisfactorily achieved.

The molecular recognition abilities of conventional adsorbents are insufficient to discriminate the complex shapes or functional groups of odorants. That is, these materials have no appropriate selectivity for odor clustering. Therefore, nanothick filters with high molecular recognition were deposited on the adsorbents using the surface sol-gel process and molecular-imprinting technique (MIT) [24–30]. Molecularly imprinted filtering adsorbents (MIFAs) were developed in these studies [31]. MIFAs were composed of the molecularly imprinted polymer (MIP) filter and a polydimethylsiloxane (PDMS) substrate. PDMSs can work as a concentrating layer to increase the quantity of odorants.

MIFAs exhibit flexible recognition ability to molecular features. They can filter volatile chemicals by the similar interactions between close molecules to recognize the mechanism of ORs. Therefore, it is speculated that the molecular information which ORs extract can also be obtained from MIFAs. Then, the partial molecular features (odotopes) recognized by ORs are expressed by various molecular parameters of chemicals. MIFAs could accomplish the indirect measurement of molecular parameters of various chemicals by appropriate selection of MIP filters and concentrating layer.

In addition, multiplex MIFAs simultaneously imprinted with different molecular sites were developed in our previous research [31]. Thus, MIFAs can be optimized for the wide selectivity to many kinds of chemicals because of the extensibility and flexibility of the molecular recognition layer. Therefore, the MIFAs-embedded sensor system makes the extraction of odotope-information of odorants and construction of odor maps close to the biological olfaction possible.

In this study, MIFAs with selective sites for various odorants were embedded with the sensor system, and artificial odor maps of odor mixtures composed of odor materials of clusters A, B, and F were constructed by classifying them into their odor-clustering attributes.

## 2. Materials and Methods

### 2.1. Chemicals

**Figure 2.**
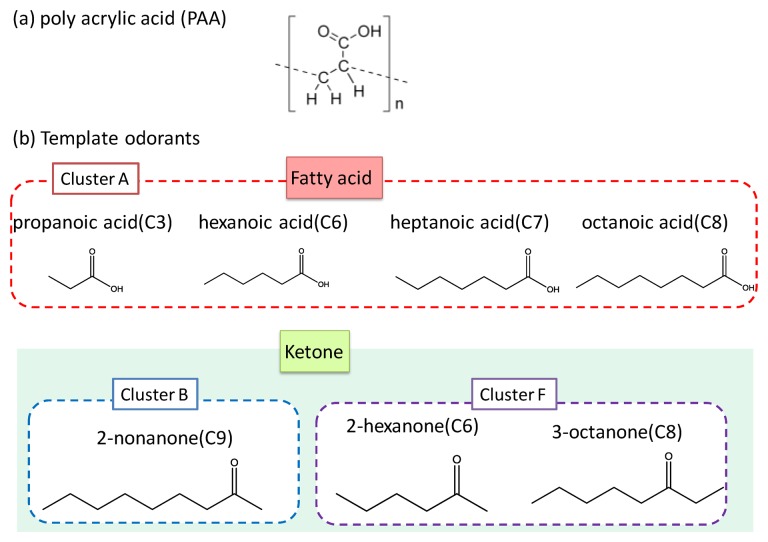
The molecular structures of PAA and template odorants. Three types of fatty acids and ketones were chosen. These odorants belong to cluster A, B, or F.

Polyacrylic acid (PAA), titanium (IV)-*n*-butoxide (Ti(O-*n*-Bu)_4_), and template odorants (propanoic acid, 2-hexanone, 2-heptanone, and 2-nonanone) were purchased from Wako, Osaka, Japan. Other templates (hexanoic acid, octanoic acid, and 3-octanone) were purchased from Kanto Chem., Tokyo, Japan. PDMS layers were fabricated using SILPOT 184 W/C of Dow Corning Toray Co., Ltd., Tokyo, Japan. All these chemicals were guaranteed reagents and were used without further purification. Molecular structures of the odorants used in this study and their clustering attributes are shown in Figure 2.

### 2.2. MIP Matrix Fabrication

**Figure 3.**
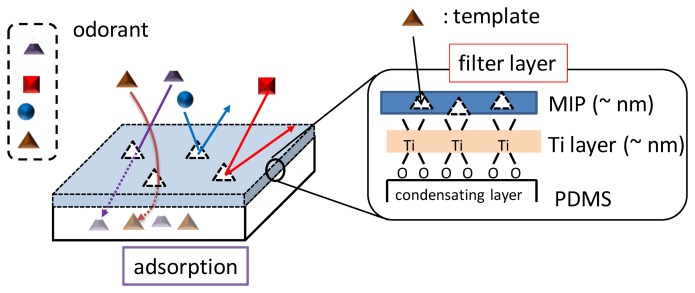
The layer structure of MIFAs sandwich with a MIP filter deposited on the PDMS adsorbents.

Figure 3 shows the overall structure of MIFAs. Odorants with similar molecular structures can be concentrated into PDMS layer through ultrathin MIP filters whose thickness was confirmed to about 7 nm by spectroscopic ellipsometry [31]. Therefore, The MIP filter layer can avoid a large amount of chemical adsorption by its nanoscale thickness. Hence, MIFAs should reduce the non-specific adsorptions.

The PDMS substrates were prepared by condensing layers of MIFAs. PDMS polymer was produced by mixing the PDMS base with a curing agent in a 10:1 *v*/*v* ratio. Bubbles generated during the mixture stirring process were removed under vacuum. Then, the PDMS assembly was cured in the oven at 150 °C for 15 min. The thickness of PDMS substrates was controlled to be 500 μm. The deposition procedure of the MIP layer on the surface of the PDMS substrate is as shown in references [23,31]; First, titanium (Ti) oxide gel layers were deposited on the hydrophilized PDMS by the surface sol-gel process through immersion in a solution of Ti(O-*n*-Bu)_4_ [32–35]. Then, the PAA/template complexes were chemically bound to the surface of the TiO_2_ film after immersing into the PAA/template solution at room temperature for 60 min. Finally, the fabricated MIFA was heated to 100 °C for more than 1 h to remove the template odorants from the MIP matrix and PDMS bulks. After removal, specific binding sites of the template odorant were molded in the assembled PAA and TiO_2_ layers. The modification of TiO_2_ monolayers and MIP filter on PDMS substrates was confirmed using spectroscopic ellipsometry and Fourier transform infrared spectroscopy [31,36].

### 2.3. GC–MS and SPME Measurement

The adsorption properties of MIFAs were examined using gas chromatography–mass spectrometry (GC–MS, QP2010-SE, Shimadzu, Kyoto, Japan) and a solid-phase microextraction (SPME) method. A Carbowax-polyethylene glycol (PEG) coated 60 μm fiber (Supelco, Bellefonte, PA, USA) was used for auto-sampling at extraction condition at 90 °C, 20 min. This fiber was selected because of the high polarity. The column used in the GC-MS was a 60 m × 0.32 mm. DB-WAX film (0.5 μm in thickness) was used for separation of various average VOCs. The analytes were desorbed from the PEG fiber in the injection port of the GC at an inlet temperature set as 240 °C. The 30 min GC measurement began with an initial oven temperature of 40 °C for 5 min, followed by a ramp of 10 °C/min till 230 °C, ended with 6 min hold. The quadrupole mass analyzer was operated in electron ionization (EI) mode, and scanned over a mass ranged of *m/z* 35–550 in full scan mode.

The selectivity of the template odorants was evaluated by measuring the absorbed quantities of a mixed odor including the templates. In this study, the adsorption amounts of MIFAs and untreated PDMS without the MIP filter were compared to estimate the selectivity of MIFAs. The mixed odor was produced using chemical solvents, desiccators, and Tedlar^®^ bag [31]. The constituent chemicals were kept in the sealed desiccator for more than 1 h to reach the saturation status. Table 1 lists the saturated vapor pressure of each molecule described by Guidechem [37] and concentration of each vapor.

The adsorption test for sample adsorbents was conducted as follows. First, MIFAs and untreated PDMS were kept in the desiccator with all the vapor constituents at the saturated status for 10 min. Second, the adsorbents were packed in a septum-capped vial after the concentration of mixed odor, separately, and placed on the tray of an automated sampler for headspace SPME (AOC-5000 Auto Injector, Shimadzu, Kyoto, Japan). Third, volatile components desorbed by heating at 90 °C were sampled using the PEG fiber for 20 min. Finally, the absorption characters of samples were analyzed using GC–MS.

### 2.4. Detection of Odors Using the Odor-Separating System

**Figure 4.**
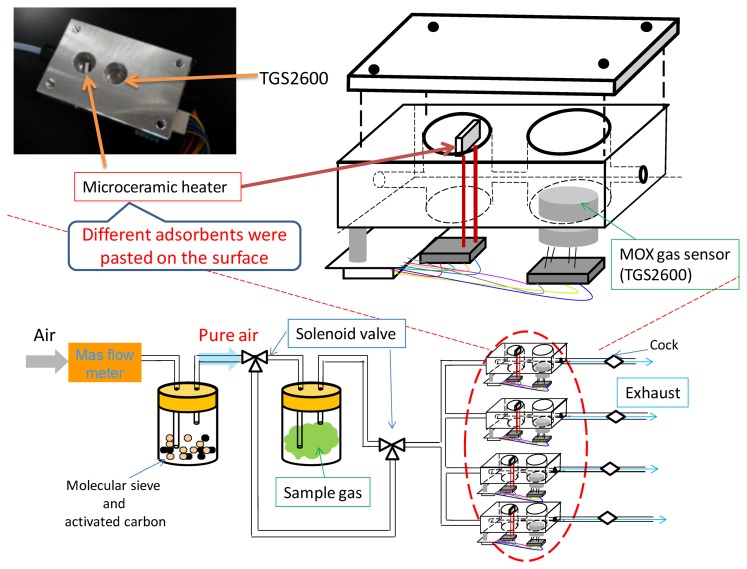
Illustration of the odor-separating system.

Odor measurements were carried out using the odor-separating system, shown in Figure 4. Four sensing cells are embedded in this system. Each cell has a microceramic heater (MS–M5, Sakaguchi E.H VOC Corp., Tokyo, Japan) in the front of a MOX gas sensor (TGS2600, Figaro Engineering Inc., Osaka, Japan). The sensing element of TGS2600 is comprised of an n-type SnO_2_ layer on an alumina substrate of a sensing chip together with an integrated heater. TGS2600s have cross-sensitivity to various chemicals, although they could not identify chemicals because of the low selectivity. The odor-separating system improved the selectivity by MIFAs.

Untreated PDMS and various kinds of MIFAs were pasted on the surface of four heaters, respectively. This system could guarantee the concentration of measured vapors into adsorbents because of the small volume of flow path (1.7 cm^3^) and low flow rate (0.15 L/min) in each sensing cell. In our previous study, the sensing system could detect and discriminate odorants in the ppb level [22]. The high sensitivity of PDMS as the concentrating materials for target VOCs was confirmed using the odor-separating system. Then, the measurement of concentration dependence of some chemicals and class identification of target VOCs were achieved [38].

**Figure 5.**
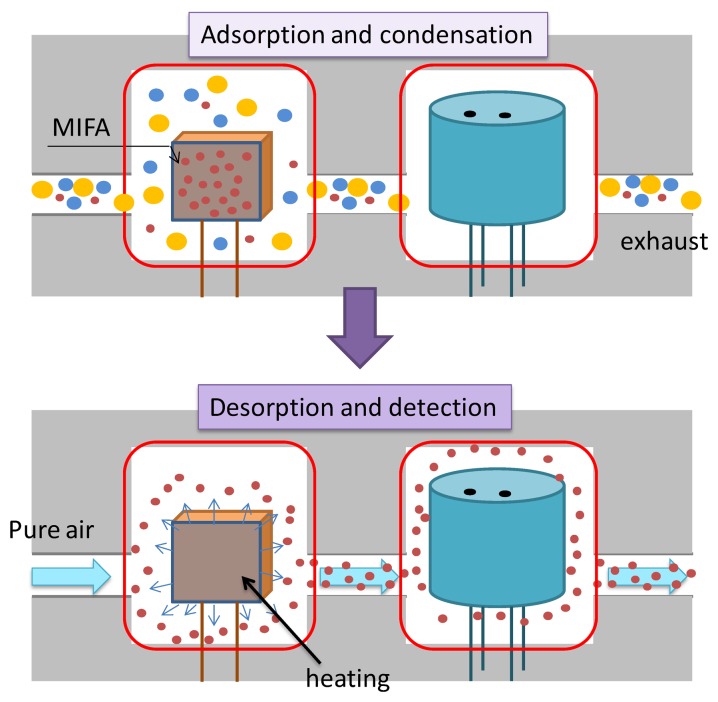
The adsorption and desorption mechanism in the odor-separating system.

An aliquot of 10 μL template bulk solution was absorbed by a cotton paper which was sealed in the gas-washing bottles. Sample vapors were attenuated with certain concentration by pure air. The concentration of sample vapors was measured as in our previous research [22]. For instance, the concentration of propanoic acid was 113 ppb. In addition, the concentration of 3-octanone injected into the system was approximately 1 ppm which were confirmed using a photoionization detector (Firstcheck+, Ion Science Ltd., Cambridge, England). It was thought that the concentration of all mixtures and odorants used in this study reached approx. 1 ppm roughly. Vapor flows were switched on for 1 min, and then switched off to flesh air flow to recover the sensor response. For desorption of the adsorbed vapors, the microceramic heater was heated to 100 °C for 200 s. Then, the linked MOX gas sensors detected and measured vapors desorbed from the adsorbents. The schematic graph of vapor concentration and separation mechanism of an odor-separating system is shown in Figure 5.

**Figure 6.**
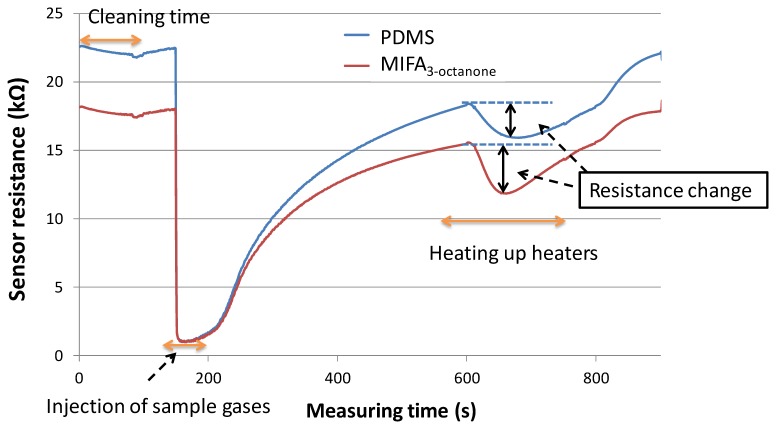
The responses of gas sensors consisting of untreated PDMS and MIFA_3-octanone_ when injecting 3-octanone into the system.

We speculated that in the vapor adsorption process, vapors having structural similarity with template sites of MIFAs were selectively adsorbed and concentrated on the PDMS substrate through MIP filters, while non-trapped odorants could be detected by the MOX gas sensors. In the vapor desorption process, the desorbed vapors from untreated PDMS and MIFAs were sent to the connected MOX gas sensor by pure air flow. Figure 6 showed the odor response of MOX gas sensors. The resistance changes induced by gas desorption from untreated PDMS and various MIFAs correspond to adsorbing properties of the MIFA and were used to evaluate the selectivity of MIFAs. The desorption of adsorbed molecules were carried out at shorter recovery time, which was thought that better sensor response can be obtained, because of the shortening of the measurement time.

### 2.5. Construction of Artificial Odor Maps

Artificial odor maps can be constructed based on the sensor responses. These maps are comprised of basic function of “ellipses” representing nine clusters in Figure 1 and “intensity” equal to sensor response of each cluster. Two coordinates *x* and *y* are coincided with odor maps of rats developed in Leon laboratory [16–19]. The equation of the coordinate (*x*, *y*) of the ellipse corresponding to cluster *N* was expressed as follows:

(1) $${\begin{array}{cc} \left[\begin{array}{c} X\\ Y \end{array}\right]=\left[\begin{array}{ll} \cos\theta_{N} & -\sin\theta_{N}\\ \sin\theta_{N} & \cos\theta_{N} \end{array}\right]\;\left[\begin{array}{c} x-x_{N}\\ y-y_{N} \end{array}\right], & \left(\frac{X}{a_{N}}\right) \end{array}}^{2}+{\left(\frac{Y}{b_{N}}\right)}^{2}=1(N=\text{A},\text{B},\cdots ,\text{I})$$

where (*x_N_*, *y_N_*), *a_N_*, and *b_N_* mean central coordinate, a semi-major axis, and a semi-minor axis of cluster *N* ellipse, respectively. Index θ*_N_* was defined as the angle between major axis of the cluster *N* ellipse and *x* axis of the map. Because the regions around active parts are gradually weakening in odor maps of rats, the activity pattern *f_N_*(*x*, *y*) in the ellipse corresponding to cluster *N* could be assumed as the following Gaussian function:

(2) $$f_{N}(x,y)=I_{\text{max}}\times R_{N}\times \frac{1}{\sqrt{2{\pi \sigma }^{2}}}\times \exp\left[-\left\{\left({\left(\frac{X}{a_{N}}\right)}^{2}+{\left(\frac{Y}{b_{N}}\right)}^{2}\right)/2\sigma^{2}\right\}\right]+{\text{I}}_{0}\;(N=\text{A},\text{B},\cdots ,\text{I})$$

where intensity index *R_N_* represents sensor response of cluster *N*, I_max_ is highest activate intensity in the map and I_0_ is an offset value of activate intensity. The standard deviation $\sigma =1/\sqrt{3}$, I_max_ = 255 and I_0_ = 30 were substituted in the Equation (2).

If an odor mixture is adsorbed into the MIFA corresponding to cluster *N*, hence, if index *R_N_* is large, the region of cluster *N* is strongly activated in the artificial map of the mix0ture; otherwise, the activity pattern of the cluster is weaker. Based on the position of each cluster in Figure 1 and activity pattern *f_N_*(*x*, *y*), gray scale images of artificial maps of odors were created. These gray images were colorized using ImageJ software (NIH, Bethesda, MD, USA).

## 3. Results and Discussion

### 3.1. MIFAs Selectivity Evaluation

The selectivity and filtration of MIFAs were evaluated by measuring the amounts adsorbed by MIFAs and untreated PDMS from a mixed odor gas using SPME and GC–MS. The gas adsorption amount of untreated PDMS was used as a reference. Our previous studies revealed the adsorbing properties of various MIFAs using SPME and GC–MS measurements [23,31]. Odorants of large molecular size cannot pass the MIP sites, whereas template and similar odorants which can pass through the MIP filter and are concentrated by untreated PDMS. These results suggest that the MIFAs with a specific site for fatty acids, ketones, and aldehydes could selectively adsorb the template odorant, and filter other odorants.

**Figure 7.**
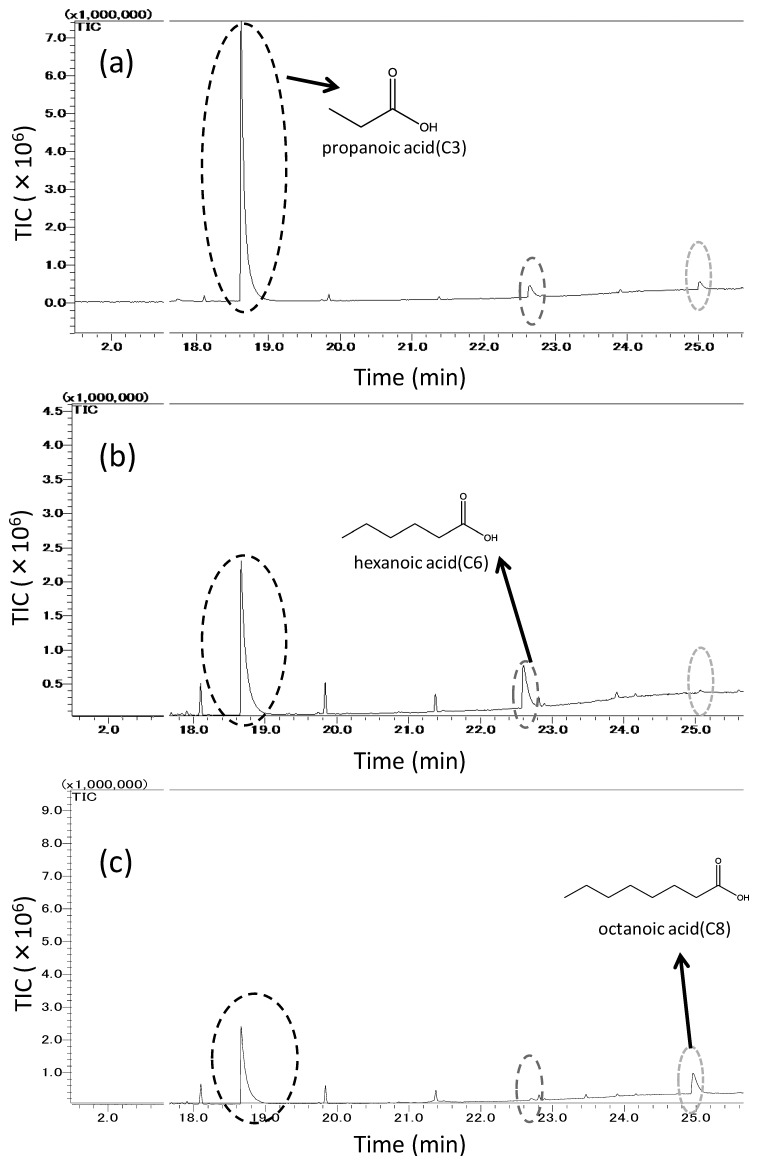
Chromatograms from 18 min to 26 min, comparison of (**a**) untreated PDMS, (**b**) MIFA_hexanoic acid_, and (**c**) MIFA_octanoic acid_ to fatty acids.

Figure 7 shows three chromatograms related to the samplings of the odorant mixture, which is consisted of propanoic, hexanoic, and octanoic acids, trapped into: (a) untreated PDMS, (b) MIFA_hexanoic acid_, and (c) MIFA_octanoic acid_. Here, we choose MIFA*_i_* to represent the MIFA with a binding site for odorant *i*. In Figure 7, the adsorption peak of propanoic acid from MIFAs decreased compared with that from untreated PDMS. It was shown that hexanoic acid was more adsorbed on MIFA_hexanoic acid_ and less adsorbed on MIFA_octanoic acid_, whereas the octanoic acid peak of MIFA_octanoic acid_ was large and that of MIFA_hexanoic acid_ was small.

**Figure 8.**
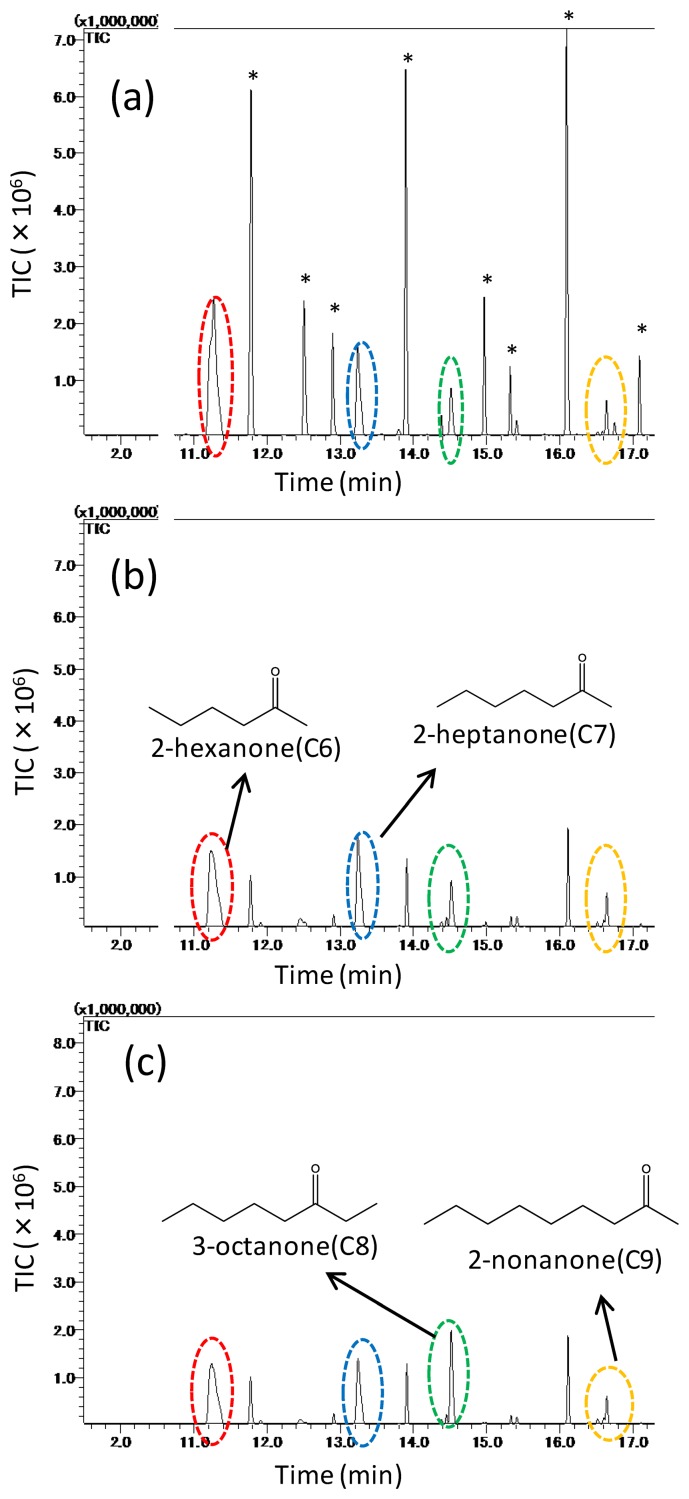
Chromatograms from 11 min to 17 min, comparison of (**a**) untreated PDMS, (**b**) MIFA_2-heptanone_, and (**c**) MIFA_3-octanone_ to ketones.

Figure 8 shows three chromatograms based on the adsorbing amounts of the mixed odor which is consisted of four types of ketones: 2-hexanone, 2-heptanone, 3-octanone, 2-nonanone, trapped on: (a) untreated PDMS, (b) MIFA_2-heptanone_, and (c) MIFA_3-octanone_. In Figure 8a, some remaining siloxane derivative peaks are shown. These peaks are attributed to the SPME fiber coating, GC-column, and the untreated PDMS as shown in Figure 8a. The eight peaks * starting from the left in the Figure 8a represent bis[(trimethylsilyl)oxy]trisiloxane, dodecamethylhexasiloxane, dodecamethylpentasiloxane, dodecamethylcyclohexasiloxane, hexadecamethyloctasiloxane, tetradecamethylhexasiloxane, tris-(trimethylsiloxy)tetrasiloxane and tetradecamethylheptasiloxane. The results show that these detected substances have little influence on the adsorption of odors. As shown in Figure 8, the MIFAs peaks of non-template odorants decreased, and the peaks of the template of each MIFA did not decrease, compared by that of untreated PDMS.

Consequently, it is believed that MIFAs represent the specific adsorption ability to the template odor in the presence of various types of chemicals. MIP filters with a high molecular sieve effect could be deposited on PDMS, and MIFAs adsorbed only the odorants that passed through the imprinted template site. However, MIFA_ketone_ shows less filtration than MIFA_fatty acid_, which may be attributed to the affinity to MIP filter of ketones or the high concentration than carboxylic groups.

### 3.2. Odor-Separating System Embedding with MIFAs

Odors were detected using the MIFAs-embedded sensor system shown in Figure 4. Because of the condensation ability of MIFAs, the system enables the measurement of odors at low concentration levels, compared to SPME/GC–MS measurement discussed in Section 3.1. Larger response amplitudes were also obtained using the MIFAs-embedded sensor system than the system using conventional adsorbent materials [22]. A sensor response index *R*(MIFA*_i_*, *j*) was defined as the resistance change of the MOX gas sensor by a sample gas *j* desorbed from MIFA*_i_* material. All indexes *R*(MIFA*_i_*, *j*) could be normalized by *R*(MIFA_0_, *j*) because the concentration of odorant *j* injected to each cell is constant. Untreated PDMS is denoted by MIFA_0_. The measured odors could be digitalized to sensor patterns based on these indexes. By comparing the sensor responses between MIFAs and MIFA_0_, the filtering degree of structurally similar odorants with the binding site can be evaluated.

**Figure 9.**
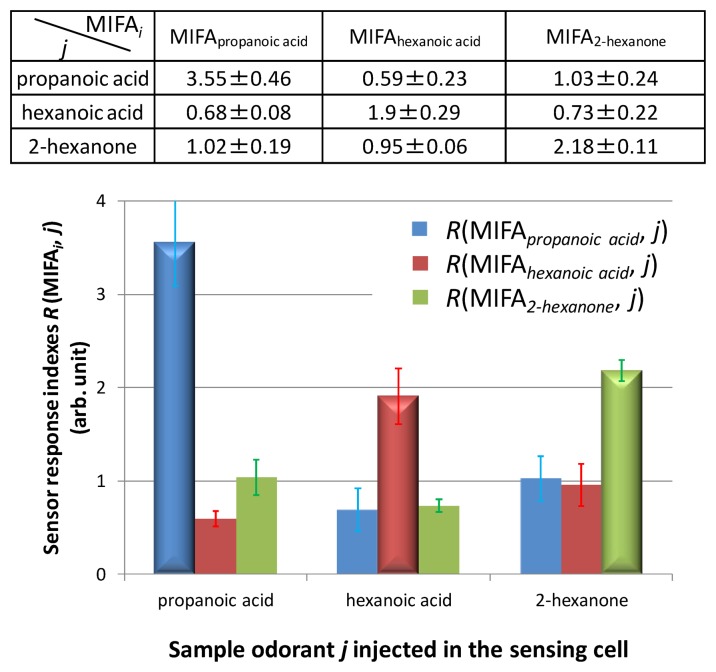
Selectivity of MIFAs with a site of various odorants obtained using the sensor system embedded with each MIFA. The vertical axis expresses indexes *R*(MIFA*_i_*, *j*) to sample gas *j*, which were normalized by *R*(MIFA_0_, *j*). 3D effect bars were used to demonstrate the sensor response index *R*(MIFA*_i_*, *i*).

First, MIFA_0_, MIFA_propanoic acid_, MIFA_heptanoic acid_, and MIFA_2-hexanone_ were pasted onto the surface of heaters in the cells. The selectivity of MIFAs was evaluated by measuring the template odorant of each MIFA using the sensor system. Figure 9 shows the adsorbing properties of MIFAs when injecting a single odor. In Figure 9, we use 3D effect bars and flat bars to demonstrate the sensor response index *R*(MIFA*_i_*, *j*) to the template vapor *i* and others, respectively.

Figure 9 shows that each MIFA selectively adsorbed the template odorant and a smaller amount of non-template odorants. The sensor response index *R*(MIFA*_i_*, *j*) corresponding to the 3D effect bars was larger than the other flat bars. As a result, it was shown that developed MIFAs exhibited molecular recognition ability toward each template. These results were similar to the adsorption results using SPME/GC–MS. Similar results were also confirmed in our previous study using a small number of sets of MIFAs [23]. Therefore, it was thought that the PDMS substrate worked as a good concentrator, and gas molecules were blocked because of the nanofiltration of MIP layers. In addition, non-specific adsorption, which was a common problem of various sensor devices, could be minimized using the measurement procedure by separating and detecting phases as well as the concentrating ability of the MIFAs.

We concluded that the selective detection and discrimination of low concentration levels of odors were confirmed by the MIFAs-embedded sensor system, hence, the odor-separating system embedded in MIFAs exhibits high sensitivity and specific odorant selectivity by the combination of MIFAs with high molecular recognition ability and MOX gas sensors.

### 3.3. Mixed Odors Detection

**Figure 10.**
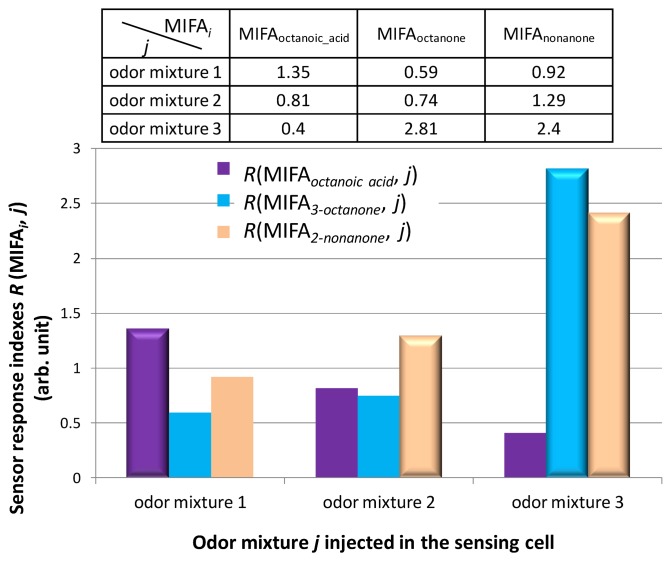
The sensor response index *R*(MIFA*_i_*, *j*) to odor mixture *j* obtained using the odor-separating system with different embedded MIFAs.

The MIFAs-embedded sensor system could simultaneously detect multiple odors. We tested three types of odor mixtures using the odor-separating system. The measured odor mixtures are shown in Table 2. The pasted adsorbents include MIFA_0_, MIFA_octanoic acid,_ MIFA_3-octanone_, and MIFA_2-nonanone_. Figure 10 shows the sensor response index *R*(MIFA*_i_*, *j*) to odor mixture *j* trapped by MIFAs, relative to MIFA_0_. The responses of MIFA_octanoic acid_ and MIFA_2-nonanone_ were maximum to the odor mixtures 1 and 2 containing octanoic acid and 2-nonanone, respectively. However, the adsorption amounts of MIFA_3-octanone_ and MIFA_2-nonanone_ were higher than one of MIFA_octanoic acid_, for the odor mixture 3 including 3-octanone and 2-nonanone. Therefore, it was suggested that the system could successfully detect template odorants selectively, and the digitization of various odor patterns was accomplished by the adsorption properties of MIFAs.

### 3.4. The Artificial Odor Map Construction

The MIFAs-embedded sensor system can extract odor information necessary for the odor-clustering map, where constituent odorants are classified into appropriate clusters. MIFAs adsorbed odorants by the interaction between molecules. Then, MIP filters can be optimized for various detection targets because of the extensibility and the flexibility of the molecular recognition layers. Therefore, it was thought that odor maps close to the biological olfaction could be roughly constructed by the adsorption ability of MIFAs, although whether MIFAs perform the direct recognition of odotopes by the same manner as olfactory receptors was not confirmed.

Furthermore, we insisted that this system realized the comprehensive detection and qualitative sensing of many types of gases by the construction of artificial odor map. We constructed an artificial odor map of individual vapors and odor mixtures based on sensor responses in Figures 9 and 10. Measured odorants could be classified into clusters A, B, or F by odor-clustering map shown in Figure 1. Table 3 lists the intensity indexes *R_N_* of each cluster, regarding propanoic acid, 2-hexanone, and the odor mixtures 1, 2, and 3. These indexes were obtained by normalizing the indexes *R*(MIFA*_i_*, *j*) shown in Figures 9 and 10 with the largest sensor response *R*_max_(MIFAs, *j*) among MIFAs. The intensity indexes of propanoic acid and 2-hexanone were calculated based on sensor responses to MIFA_propanoic acid_ and MIFA_2-hexanone_ in Figure 9, respectively. *R*_A_, *R*_B_, and *R*_F_ of odor mixtures in Table 3 corresponded to sensor responses to MIFA_octanoic acid_, MIFA_2-nonanone_, and MIFA_3-octanone_ in Figure 10, respectively. We assumed it zero for other indexes *R_N_* because MIFAs corresponding to cluster were not embedded in the system.

**Figure 11.**
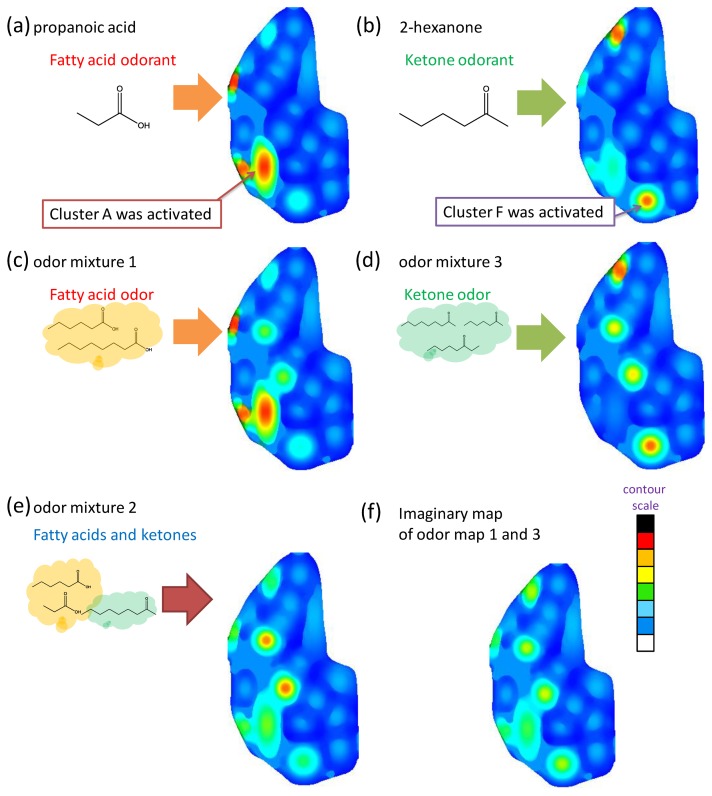
The artificial odor maps of (**a**) propanoic acid, (**b**) 2-hexanone, (**c**) odor mixture 1 (fatty acids), (**d**) odor mixture 3 (ketones), and (**e**) odor mixture 2 (fatty acids and ketones) based on sensor response shown in Figures 9 and 10. The map (**f**) indicates the imaginary odor map obtained by overlaying images of (a) and (b).

The artificial odor maps of these sample odors were constructed by measuring: (a) propanoic acid, (b) 2-hexanone (c) fatty acid binary odor, (d) ketone ternary odor, and (e) fatty acid and ketone mixture using the MIFAs-embedded sensor system, respectively (Figure 11). Then, they were divided into categories on the basis of the analysis of the image features of approximately 400 odor maps developed by the Leon laboratory [16–19]. The color strength scale of each cluster is proportional to the sensor response represented in Table 3 as the contour scale in Figure 11.

As shown in Figure 11a,b, the areas corresponding to propanoic acid and 2-hexanone, which could be classified in clusters A and F, were activated by MIFA_propanoic acid_ and MIFA_2-hexanone_, respectively. Naturally, these maps coincided with the physiological information of the odor maps developed in the Leon laboratory. The construction of odor maps close to the biological olfaction was successfully examined using the MIPAs-embedded sensor system. In Figure 11c, the area of cluster A that corresponds to fatty acids was strongly activated, whereas the area of other clusters was either weakly stimulated or not stimulated, based on the *R_N_* to odor mixture 1. Figure 11d indicates that the corresponding parts of cluster B and F were activated and gradually weakened.

Here, ketone odorants are classified in different clusters by the carbon chain number, e.g., 2-nonanone and 3-octanone are categorized into clusters B and F, respectively. Therefore, the difference of odorants belonging to different clusters and with the same functional groups were successfully discriminated by artificial odor maps constructed by using the MIFAs-embedded sensor system. Odor mixtures were expressed by their odor-clustering attributes on the map, and the map simultaneously activated some clusters as the biological odor map.

Figure 11f displays the composite map constructed with artificial odor maps of fatty acid odor (Figure 11c) and ketone odor (Figure 11d). Comparing the odor map obtained by measuring fatty acids and ketones (Figure 11e), there exists image similarity. Lin *et al.* revealed that an activity pattern of an odor is equivalent to the overlaid map accumulating the response elicited by each constituent molecule in the biological olfaction [39]. This was also demonstrated by higher similarity between the propanoic acid map (Figure 11a) and fatty acids map (Figure 11c). Then, the 2-hexanone map (Figure 11b) represents partial activity patterns of the overlaid map (Figure 11d). Therefore, it is believed that the constructed maps are close to the internal expressions of biological olfactory sensing and may contain the same physiological information about the quality of an odor. As a result, the difference between odors was visually presented by constructing an artificial odor map. Therefore, the MIFAs-embedded sensor system enables the visualization and classification of various odors.

In this study, we investigated the odors belonging to cluster A, B, and F. Comprehensive detection and appropriate classification of odors can be accomplished as in biological olfaction by covering all clusters shown in Figure 1, through adding the cell numbers in the sensor system and various MIFAs. For example, multiplex MIP filters with different imprinted sites were also developed in previous research [31]. Adsorbents which selectively concentrate odorants belonging to each cluster should be developed by designing of adsorption properties of MIFAs.

## 4. Conclusions

In this study, a selective site for a variety of odorants could be formed on MIFAs and the precise molecular recognition ability of MIFAs was confirmed based on the selectivity results of the SPME and GC–MS methods. The sensor responses to single vapors and odor mixtures were successfully measured using the MIFAs-embedded sensor system. Based on the responses, artificial maps of these odors were constructed. Consequently, this system enables the optimal classification of the odor information by visualizing the odors.

We have successfully developed MIFAs with a binding site for fatty acids, ketones, and aldehydes using PAA or peptides [36,40]. However, sites for complex odorant groups cannot be formed with our MIP method. Therefore, it is necessary to apply another MIT for the development of MIFAs with a specific binding site for odorants belonging to other clusters, e.g., aromatic, alcohols, hydrocarbons, and cluster MIFAs, which can simultaneously absorb odorants belonging to the same cluster.

## Figures and Tables

**Table 1.** The saturated vapor pressures of constituent odorants and concentrations of each vapor generated in desiccator. Each saturated vapor pressure is presented on the websites of Guidechem. The concentrations were calculated according to the saturated vapor pressures.

**Chemicals**	**Saturated Vapor Pressure (mmHg)**	**Concentration of Each Vapor Prepared in Glass Chamber (ppm)**
Propanoic Acid (C3)	3.53	4,560
Hexanoic Acid (C6)	0.435	560
Octanoic-Acid (C8)	3.71 × 10^2^	50
2-Hexanone (C6)	11.6	15,000
2-Heptanone (C7)	3.85	5,990
3-Octanone (C8)	1.35	1,745
2-Nonanone (C9)	0.55	710

**Table 2.** Constituent odorants of each odor mixture.

**Odor Mixture**	**1**	**2**	**3**
**Constituent Odorants**	octanoic acid + hexanoic acid	propanoic acid + hexanoic acid + nonanone	Heptanone + octanone + nonanone

**Table 3.** Intensity index *R_N_* of cluster *N* calculated based on *R*(MIFA*_i_*, *j*) to odor mixture *j* shown in Figure 10. The indexes *R_N_* were normalized by the largest sensor response among MIFAs.

**Cluster Number**		**A**	**B**	**F**
Sensor response index R*_N_* of	Propanoic acid	1	-	0.29
	2-Hexanone	0.47	-	1
	Odor mixture 1	1	0.68	0.44
	Odor mixture 2	0.63	1	0.58
	Odor mixture 3	0.14	0.86	1
